# Best practices for community-based overdose education and naloxone distribution programs: results from using the Delphi approach

**DOI:** 10.1186/s12954-022-00639-z

**Published:** 2022-05-28

**Authors:** Lynn D. Wenger, Maya Doe-Simkins, Eliza Wheeler, Lee Ongais, Terry Morris, Ricky N. Bluthenthal, Alex H. Kral, Barrot H. Lambdin

**Affiliations:** 1 RTI International, 2150 Shattuck Ave, #800, Berkeley, CA 94704 USA; 2 Remedy Alliance, Chapel Hill, NC USA; 3 Keck Medicine, Department of Population and Public Health Sciences, University of Southern California, Los Angeles, USA; 4 University of California, San Francisco, USA; 5 University of Washington, Seattle, WA USA

## Abstract

**Background:**

Opioid-related overdose deaths have surged in the USA over the last two decades. Overdose fatalities are preventable with the timely administration of naloxone. Syringe service programs (SSP) have pioneered community-based naloxone distribution through overdose prevention and naloxone distribution (OEND) programs. There is a dearth of information with regards to best practices for community-based OEND.

**Methods:**

We utilized a modified Delphi approach to develop a set of best practices for OEND delivery. Starting with an initial list of best practices, we engaged 27 experts, in the field of OEND programming who reviewed, made recommendations for changes, and assigned a priority to each best practice.

**Results:**

Two rounds of input resulted in a final list of 20 best practices organized into four categories. The mean priority scores ranged from 1.17 to 2.17 (range 1 to 3). The top 5 ranked best practices were ensuring that SSP participants have low barrier, consistent, needs-based access to naloxone and that there is ample naloxone available within communities. While the remaining fifteen best practices were deemed important, they had more to do with organizational culture and implementation climate.

**Conclusions:**

Increasing community-based OEND delivery is essential to reduce opioid overdose deaths; however, it will be insufficient to add programs without an eye toward quality of implementation and fidelity to the model upon which the evidence is based. This list of best practices summarizes the consensus among OEND experts and can serve as a tool for SSPs providing OEND programming to improve services.

## Background

Opioid-related overdose death rates have surged in the USA, more than quadrupling over the last two decades [1]. In recent years, the accelerated rise in overdose deaths, which worsened during the COVID-19 pandemic [2], has been due, in part, to the introduction of illicitly manufactured fentanyl into the US drug markets [3–5]. Nearly 108,000 people died in the USA of drug overdoses in 2021, nearly 50% higher than in 2019, and approximately two-thirds of those deaths involved fentanyl or another synthetic opioid [2]. The acceleration of opioid-related overdose deaths since the start of the pandemic is due to disruptions in substance use treatment, harm reduction services, and other resources that provide people who use drugs (PWUD) support for their basic needs [6, 7]. In addition, overall increases in substance use [8], interruptions and changes in the illicit drug supply [9], and social distancing measures implemented to prevent the spread of COVID-19 [7, 10] put PWUD at increased risk of opioid-related overdose. Naloxone is an evidence-based intervention that reverses opioid-induced respiratory depression which can lead to a fatal overdose [11]. Yet, access to naloxone remains insufficient and inequitable [12–14].

Syringe service programs (SSPs) have been the mainstay for community-based HIV/HCV prevention efforts for people who use drugs (PWUD), and they reach people who typically face inadequate access to evidence-based interventions. SSPs, which provide access to and disposal of sterile syringes and injection equipment for PWUD, remain the cornerstone of public health efforts to prevent HIV among PWUD [15]. These programs also pioneered the delivery of naloxone for PWUD through overdose education and naloxone distribution (OEND) programs [16–19].

SSPs are ideal settings for OEND because their staff are culturally competent in providing services for PWUD, who already engage with and trust these organizations to care for their health. In addition, SSP participants are typically at high risk for either experiencing or witnessing an opioid overdose. Engaging SSP participants in OEND is critical; fentanyl-involved overdoses occur rapidly, and naloxone must be administered quickly to prevent mortality [14, 20]. SSPs first integrated OEND in 1996 [21–23]. Essentially, OEND programs train laypeople—people who use drugs, family members, peers—as prospective responders in overdose events by providing access to naloxone and training for its delivery [16, 18]. A strong body of research has shown that OEND can be highly effective and cost-effective at preventing opioid overdose mortality [24–27].

In response to the surge in opioid-related overdose deaths, every state as well as the District of Columbia adopted legislation to facilitate OEND service implementation [28, 29]. By 2019, 94% of SSPs across the country had incorporated OEND services into their program, a sizeable jump from 56% in 2013 [18, 30, 31]. Furthermore, the Centers for Disease Control and Prevention has made recommendations to expand OEND through public health departments and community-based organizations, healthcare providers, first responders, and other harm reduction organizations [5].

While the increase in the number of SSPs providing OEND services has been sizeable, many SSP OEND programs are operating within larger agencies, health departments, or political environments that are new to or opposed to harm reduction approaches to health promotion among PWUD. Programs often encounter problems obtaining funding for staff, supplies, and operations, locating program sites, recruiting, onboarding and training appropriate staff, and maintaining positive relationships with the larger community and law enforcement [31].

While best practices have been established for the delivery of SSP services in the USA and Canada [32, 33], and the WHO has developed guidelines for OEND delivery [34], nuanced recommendations for the best practices of community-based OEND delivery within SSPs specific to the US context do not yet exist. Best practices are a series of recommendations for service delivery based on practice-based evidence from well-established programs combined with the best available scientific evidence [33, 35]. The objective of identifying best practices is to avoid wasting resources or reinventing the wheel by learning from others under comparable circumstances [35]. Best practice recommendations identify targets for program and systems-level improvements and provide benchmarks to evaluate programs.

While the proliferation of OEND services is vital, understanding how SSPs can deliver high-quality OEND services is paramount to addressing the nation’s opioid overdose crisis. While research has documented some evidence-based approaches [36–39], the current evidence base is not yet comprehensive. Defining and implementing best practices for OEND services within SSPs is critical to ensure a productive climate for implementation, fidelity to the model to which the evidence is based, and ultimately achieve implementation effectiveness—defined as consistent, effective use of naloxone during overdose events. To fill this gap, we conducted a Delphi study with a panel of experts in OEND programming to leverage over 20 years of OEND implementation experience and define best practices for OEND delivery at SSPs grounded in practice-based evidence.

## Methods

We utilized a modified Delphi approach to develop a set of best practices for OEND delivered at SSPs [40–43]. Our objective was to identify the current state of knowledge and practices with regards to SSP-based OEND delivery that could strengthen community-based responses to reduce opioid-involved overdose deaths. At its core, the Delphi approach is an iterative communication process designed to critically examine a particular topic with a diverse group of experts [41, 43]. It is often used with topics where empirical evidence is limited [44] and is particularly useful when the subject being studied is complex and requires intuitive interpretation of evidence [43]. A recent review of the Delphi technique within the health sciences identified that most studies included 2–3 rounds [42].

We began the Delphi process with an initial list of possible best practices developed by two experts in the field of OEND (authors MDS and EW)—each with more than two decades of experience working in and providing support for SSPs implementing community-based OEND. In addition, they had previously developed the only implementation guide for OEND for community-based organization and continue to operate a national network of OEND leadership and experts to support US-based OEND delivery. Our study team organized an initial list of 19 best practices with definitions categorized in three domains. Next, we identified and enrolled participants who were experts in the field of OEND delivery and carried out two rounds of inquiry for the Delphi study.

### Participant selection

We identified an initial list of 27 potential expert study participants from diverse locations across the USA, representing people working in rural, urban, and suburban areas. The sample included people in paid and volunteer leadership and direct service positions in SSPs, OEND researchers, people who work in state or local health departments, and people who use drugs who deliver and access SSP/OEND services. All individuals had prior and current experience delivering OEND programming in community-based settings, and people with lived substance use experience were represented in each of the expert categories. Importantly, experts currently accessing SSP-based OEND services were included; after we interviewed people working in SSPs, we asked them to refer people with lived experience who were interested in and felt comfortable with participating in an in-depth interview over Zoom. Twenty-seven experts participated in Round 1 of the DELPHI process; 12 people in leadership positions in SSPs, 2 researchers, 6 people who work in health departments, and 7 people who were current SSP participants. Each expert was paid $50 to compensate them for their time spent participating in the study. All participants engaged in a verbal informed consent process prior to their interview. All study procedures were approved by the institutional review board of RTI International.

Recruitment of potential participants began in April 2019, and data collection was completed in August 2019. Potential participants first received a recruitment phone call briefly describing the study and then a follow-up email with details about the study, a copy of the draft best practice document, and a request to participate in the Delphi process.

### Delphi rounds and procedures

#### Round 1

For the first round, 27 experts took part in a one-time hour-long in-depth interview, conducted over Zoom one-on-one with one of two interviewers trained in qualitative interviewing techniques (authors LO and TM). During the in-depth interview, each of the best practices was reviewed with participants. Once reviewed, participants were asked the following questions: What are your thoughts on these best practices? What other best practices would you add? Which best practices do you not agree with? What changes would you make to these best practices? What changes would you make to the grouping of best practices? If a person indicated a change, follow-up questions were asked with regards to whether the expert disagreed with the concept of the best practice, the language being used, and how to address those concerns. Interviews were recorded, and during the call, interviewers took notes about participants’ responses and any recommendations made by the experts, such as changes in wording, additional concepts, redundancies, or deletions. At the end of the call, the interviewers briefly described the procedures for Round 2 and let participants know they would be receiving a follow-up email with instructions in 4–6 weeks.

Following the interviews, the research team met to review the feedback and incorporate it into the next draft of the best practices document. For this process, a facilitator (author LDW) guided the discussion among the study team members. Initially, interviewers (authors LO and TM) reported information they gathered for a particular best practice. After reviewing and coding the feedback from the experts for each best practice, the study team utilized an inductive analysis approach to aid in the understanding of the data through the development of summary themes and categories from the raw data [45]. To this end, the study team reviewed and discussed the responses and collectively made decisions to modify the existing language of a best practice, divide them into more than one best practice, or move them into a different category. A consensus decision-making process was used; if an individual of the study team had any concerns with a decision, the team continued to discuss and tailor until all concerns were addressed. We did not encounter any situations where experts provided completely opposing or irreconcilable views with regards to a best practice.

#### Round 2

After the research team revised the best practices based on round 1 interviews, emails were sent instructing participants to review the revised document and reply to the email with the following instructions: (1) provide wording or language suggestions for each of the specified best practices; (2) inform the research team if they found that a previous suggestion had not yet been sufficiently incorporated into the best practice; and (3) assign a priority score of 1, 2, or 3 to each of the best practices. Priority scores were defined as follows: A score of 1 means that achieving this best practice is critical, should be focused on now, and will have the highest impact at reducing opioid overdose deaths in the community; a score of 2 means achieving this best practice is important, should be focused on soon, and will have a medium amount of impact at reducing opioid overdose deaths in the community; and a score of 3 means achieving this best practice is less important, should be focused on later, and will have less impact at reducing opioid overdose deaths in the community. We calculated the mean priority score for each best practice; then, we rank-ordered the best practices from lowest (highest priority score) to highest (lowest priority score).We initially planned to have a third round of the Delphi process. However, the panel of experts recommended making very few changes to the best practices during round 2, obviating the need for a third round.

People who were current SSP participants were encouraged to participate in more than one round of the study prior to enrollment; however, they were sent their incentive payment immediately after completing Round 1, while the rest of the participants were sent their incentive payment after completing Round 2. Although we knew that this could reduce participation in multiple rounds, this decision was made prior to recruitment, when we anticipated conducting three Delphi rounds, as the research team knew it was important to provide financial incentive to SSP participants as soon as possible, thought it would be difficult getting in touch with them multiple times and did not want to burden participants with multiple rounds of data collection.

## Results

### Round 1

All experts agreed that all the best practices were important components of effective OEND programming and did not suggest eliminating any of the best practices that were presented. Participants felt that an establishment of best practices such as these were not necessarily mandatory but could be used as guiding principles for program implementation. The best practices are organized into the following four categories: staff training and support, naloxone saturation and supply, culturally appropriate services, and grounded in harm reduction. The final best practices and their definitions are found in Table 1.

**Table 1:** Final compilation of SSP-based OEND best practices with accompanying definitions

Staff training and support
*1. Proactive engagement*
SSP staff /volunteers proactively ask participants if they would like naloxone and overdose prevention education
*2. Needs-based training*
OEND trainings can be completed in as little as 5 min and follows the participant’s needs
*3. Follow-up support for vicarious trauma*^¥^
SSPs provide support for overdose prevention educators to address experiences of vicarious trauma
*4. Follow-up support for burnout*^‖^
SSPs provide support for overdose prevention educators to address burnout
*5. Onsite overdose protocol established*
SSP has an onsite overdose protocol, and staff/volunteers are trained to respond to an overdose during service provision
*6. Training of trainers*
Overdose prevention educators are trained in:
• Engagement, counseling, and listening skills
• Delivering health education for safer drug use, overdose prevention/response, and naloxone administration
• Working with participants to develop personal overdose prevention plans
• Supporting participants with experiences of witnessing overdoses and administering naloxone
• Providing referrals to health, substance use, and social services

Naloxone saturation and supply
*7. Needs-based naloxone distribution*
Naloxone distribution is based on participant’s needs and the needs of their community. *
*8. Naloxone is accessible*
Overdose prevention trainings and naloxone distribution is provided at all syringe access service sites and during community-based outreach
*9. Sufficient naloxone supply*
Naloxone inventory is accessible to SSP staff/volunteers, and the program has enough naloxone to not run out or need to ration to participants for the next 3 months
*10. Naloxone saturation*
Annually, SSPs distribute 20 times or more the number of naloxone doses as the number of opioid overdose death in the previous year (or for the most recent year overdosed death data are available)
*11. Option to choose naloxone administration modality*
Participants can choose intranasal or intramuscular naloxone, based on their preferences

Culturally appropriate service
*12. Involvement of people who use drugs*^†^
People who use drugs deliver overdose prevention education, distribute naloxone, contribute to naloxone programming, and provide oversight of program activities
*13. Lay Person Naloxone Team*
SSPs utilize non-medical staff/volunteers to provide overdose prevention education and naloxone distribution
*14. Overdose response information and education materials offered*
Educational materials regarding overdose risk and response, such as pamphlets, posters, palm cards, and/or Web-based resources, are available to SSP participants
*15. Outreach and marketing conducted*
SSP publicizes naloxone programming by distributing information through flyers, pamphlets, posters, and social media; building community partnerships; and conducting outreach in the community

Grounded in harm reduction
*16. OEND program is grounded in harm reduction principles*^‡^
SSP staff/volunteers are trained and supported to offer services grounded in harm reduction principles
*17. Low threshold services*
SSP offers walk-in services without the need to make appointments
*18. Naloxone at no cost*
Naloxone is distributed by the SSP and is free
*19. Anonymous Service Delivery*
Participants are not required to provide personal information or identification to receive naloxone
*20. Only essential data are collected*
SSP only collects essential information from participants for program improvement, reporting, or advocacy

^¥^*Vicarious trauma*—the negative changes that happen to people over time as they witness and engage with other people’s suffering and need that can leave them feeling numb, disconnected, isolated, overwhelmed, and depressed. Over time, this process can lead to changes in your psychological, physical, and spiritual well-being

^‖^*Burnout*—the feeling of physical and emotional exhaustion due to stress from working with people under difficult or demanding conditions

^*^Includes secondary naloxone distribution (peer-to-peer distribution)

^†^These are active program participants

^‡^According to Harm Reduction International, harm reduction is a set of evidence-based practices that minimize the negative impacts of drug use and drug policies. Harm reduction is fundamentally grounded in principles that protect human rights and improve public health and incorporates a wide array of approaches designed to meet people where they are and offer opportunities for people to improve their health and well-being. Harm Reduction Coalition has designated a set of eight principles central to harm reduction practice which can be found here: https://harmreduction.org/about-us/principles-of-harm-reduction/

The expert panelists recommended modifying 16 of the original 19 best practices during Round 1 of the Delphi study. These changes included disentangling certain best practices that included multiple concepts, combining best practices that appeared repetitive, moving best practices from one category to another, renaming categories to better communicate the overarching concept, adding a new best practice, and editing the definitions of best practices to improve clarity. For example, the best practice “Follow-up support for Burnout and Trauma” was split into two best practices: (1) “Follow-up support for Burnout” and (2) “Follow-up support for Vicarious Trauma.” Experts encouraged splitting this best practice into two because burnout and vicarious trauma, while related, were different experiences that need to be recognized as such and required different types of support. In addition, the panelists recommended the inclusion of detailed definitions of these two processes to clarify their meaning. These definitions can be found at the bottom of Table 2.

**Table 2:** Best practices in order priority ranking

Best practice	Rank
Naloxone is accessible	1
Needs-based naloxone distribution	2
Sufficient naloxone supply	2
Low threshold services	2
Naloxone at no cost	2
OEND program is grounded in harm reduction principles	6
Naloxone saturation	7
Involvement of people who use drugs	8
Proactive engagement	9
Needs-based training	9
Lay Person Naloxone Team	9
Anonymous Service Delivery	12
Only essential data are collected	12
Training of trainers	14
Onsite overdose protocol established	15
Overdose response information and education materials offered	15
Outreach and marketing conducted	17
Option to choose naloxone administration modality	17
Support for vicarious trauma	17
Support for burnout	20

Although none of the experts suggested removing any of the best practices, we were encouraged to add the best practice “Only essential data are collected.” The rationale for including this best practice centers on respecting a participant’s privacy and time and ensuring data collected directly contribute to program improvement. This best practice would encourage programs, funders, and policymakers to be thoughtful and deliberate about the data they collect or require for reporting.

### Round 2

We received 17 responses to the second round with very few recommended changes to the document. Two of the experts commented on the best practice that was originally labeled “OEND Saturation of Syringe Access Program Participants,” which was originally defined as “SSP is reaching 90% or more of participants with overdose prevention education and naloxone distribution.” The experts inquired about how we arrived at 90% and whether this specificity could be based in research findings. Following this, we reviewed the literature to see whether there were defined levels of naloxone saturation that were found to reduce levels of opioid overdose mortality. From this review, we found a study showing that opioid overdose deaths in Scotland decreased by half once the program distributed 20 times as many take home naloxone kits as opioid-related deaths in the prior year [37]. We clarified the intention of this best practice by renaming it “Naloxone Saturation” and defining it based on these research findings.

The mean priority scores assigned to each best practice ranged from 1.17 to 2.17 with very little variation between the highest ranked and lowest ranked best practices. Table 2 lists the best practices as they were ranked by the experts during Round 2.

## Discussion

The surge in opioid-related overdose rates in the USA over the last two decades [1, 2, 6–8] combined with insufficient and inequitable access to naloxone across the country [12, 14] has brought to light the urgent need for the development and implementation of best practices for OEND programming within SSPs. To this end, we utilized input from a panel of experts in the area of OEND programming to identify and rank best practices for OEND delivery from SSPs.

The final best practices (see Table 1) were designed as fundamental principles for OEND implementation, to provide SSP participants, staff, health departments, policymakers, funders, and surrounding communities the guidance they need to reduce opioid overdose mortality. These best practices can be used as a resource for SSPs to understand and improve their implementation quality. While they are specific to SSP-based OEND, other programmatic settings could also implement them. When employed, these practices are intended to influence all facets of OEND programs, including individual encounters with program participants, the underlying values of the organization, and system-level changes with the goal of increasing implementation quality and quantity of naloxone distribution, system-level changes to increase resources and reduce barriers, to prevent opioid overdose fatalities.

The five best practices that were ranked highest by the panel of experts were as follows: (1) naloxone is accessible, (2) needs-based naloxone distribution, (3) sufficient naloxone supply, (4) low threshold services, and (5) naloxone at no cost. Implementation of these best practices was ranked highest by the panel of experts as they are related to making sure that people have easy and consistent access to naloxone and that there is ample naloxone available within communities to address opioid-involved overdoses. Ranked lower, the remaining fifteen best practices were considered important, but not critical, for SSP-based OEND programming. Notably, these best practices had more to do with the organizational culture and less to do with the urgent imperative of getting naloxone into the hands of people who are most likely to witness and potentially respond to an overdose.

The best practices focused on support and training for burnout and vicarious trauma were deemed important but ranked last by the panel of experts. It could be that ranking reflects the ways in which these issues have historically been addressed by SSPs and where things stand in the response to the overdose crisis. Programs have been forced to prioritize acquiring and distributing life-saving supplies leaving little time and resources to focus on other elements that can be vital for the long-term stability of SSPs and their staff [39]. Yet burnout and vicarious trauma are very real hazards faced by SSP staff, volunteers, and participants. Reports of stress, trauma, and grief are commonplace for those responding daily to overdoses and living and working in a culture of punitive welfare systems, poverty, and the war on drugs [46–48]. There has been increasing attention toward the need to address and mitigate the role of these hazards through individual-, community-, and policy-level interventions [48–52]. Although ranked lower by the panel of experts, it is imperative to include support for burnout and vicarious trauma as best practices for SSP-based OEND programs to highlight how these issues are having an impact on program staff, participants, and communities.

We began our modified Delphi study by starting with a list of best practices developed by people with years of experience in the field of overdose prevention. Next, we engaged a panel of experts, including people with lived experience of drug use, to review and make recommendations for changes which resulted in an informed list of best practices for OEND programming within SSPs. The panel of experts did not recommend removing any of the best practices. Utilizing their expertise in the field they helped us refine and define a list of best practices that focus on ensuring that all SSP participants are provided access to a sufficient and consistent supply of naloxone which over time could improve efforts to prevent opioid overdose deaths. Through the process of rating the best practices and the development of a composite score, we learned that all the best practices were deemed either critical or important by the panel of experts to have an impact on reducing opioid overdose deaths in the community. The composite scoring did show that some of the best practices were rated slightly higher in priority than others, but none fell into the “less important should be focused on later and will have less impact at reduction opioid overdose death” category.

While we believe it is essential to define and implement best practices for OEND delivery among SSPs, we recognize that these programs do not exist in a vacuum. SSPs exist within a context of the racialized drug policies that lead to the criminalization of drug use and drug users and are often underfunded and under-resourced harm reduction programs [47]. Geographic differences in the socioeconomic make-up of communities, regional variations in the drug supply [53], and overdose rates [54, 55] make some best practices more relevant than others depending on the location of SSPs in the USA. Thus, implementation of best practices will look different across programs and locales due to geographic and demographic differences. This set of best practices were devised with these constraints in mind; thus, even though the goal of the best practices is to provide guidance to programs, funders, and policymakers to implement high-quality SSP-based OEND programming, we acknowledge that in areas where funding is scarce or policies limit service delivery, these best practices could be impossible to achieve. We also recognize that although the overall goal of this study was to develop SSP-specific best practices for OEND programming, community-based settings that are distributing naloxone and are not SSPs should strive to adhere to these best practices because SSPs are the setting where OEND was developed and evaluated. Deviation from the SSP model may not achieve similar protectiveness against fatal opioid overdose.

Although the final list of best practices is a useful tool for helping SSPs improve naloxone implementation, this study has the following limitations. The sample of experts that participated in this study was relatively small, and their opinions may not represent the opinions of all experts in the area of OEND programming. Nonetheless, it was drawn from diverse locations across the USA and represent people working in rural, urban, and suburban areas. Second, SSP participants were invited to participate in both rounds of the survey. However, they were told they only had to complete one round of the study to receive their incentive payment, while the other participants were paid after they completed both rounds. Although the study team knew this could impact participation in subsequent rounds of the study, it was more important to pay these participants as soon as possible. In the end, only one of the seven SSP participants who participated in Round 1 completed Round 2. While the study team made this decision to minimize burden on SSP participants, future studies should design ways to engage SSP participants throughout multiple rounds to the extent possible. Examples of how one could ensure better inclusion in the future would be to pay individuals for participation in each round of the study, making it increasingly worthwhile to complete the study or oversample SSP participants in early rounds to account for a larger dropout rate. Third, our study focused only on SSP-based OEND services. We believe that extending these best practices to other programmatic settings will reduce overdose deaths; however, it will require thoughtful consideration. For example, most of the best practices could easily apply to other community-based settings, but some such as “Anonymous Service Delivery” or “Lay Person Naloxone Team” might not apply to OEND services delivered from jails or emergency departments. Fourth, the priority scale that was provided to the experts had a very small range [1–3] and little variation was observed in the scoring. We may have seen more variation in the results if we had presented the experts with a larger scale. Finally, issues related to the impact of the toxic drug supply and OEND programming did not emerge in this study. Future initiatives to define best practices for SSP-based OEND must consider the changing toxicity of the drug supply.

## Conclusions

Scaling up SSPs is essential to reduce opioid overdose deaths and sustain community-based OEND programming. However, it is not sufficient to simply add additional programs without an eye toward their quality of implementation and fidelity to the intervention upon which the evidence is based. The best practices devised by this group of experts can serve as a tool for SSP-based OEND programs to improve upon their services. This necessitates programs have sufficient funding for supplies, staffing, and infrastructure, provide staff training and support, offer culturally appropriate services and to make sure all services being provided are grounded in harm reduction. To do this, it is essential that people with lived experience, who are already engaging in peer-based harm reduction, are included at all stages of program implementation. It is also imperative that public funding for SSP-based OEND programming, policy change, and technical support for implementation is available and accessible to all programs.

## Data Availability

The datasets used and/or analyzed during the current study are available from the corresponding author on reasonable request.
